# Connecting the Dots of Social Robot Design From Interviews With Robot Creators

**DOI:** 10.3389/frobt.2022.720799

**Published:** 2022-06-02

**Authors:** Patrícia Alves-Oliveira, Alaina Orr, Elin A. Björling, Maya Cakmak

**Affiliations:** ^1^ Paul G. Allen School of Computer Science and Engineering, University of Washington, Seattle, WA, United States; ^2^ Department of Human-Centered Design and Engineering, University of Washington, Seattle, WA, United States

**Keywords:** social robots, market studies, product design, qualitative research, domain experts

## Abstract

Despite promises about the near-term potential of social robots to share our daily lives, they remain unable to form autonomous, lasting, and engaging relationships with humans. Many companies are deploying social robots into the consumer and commercial market; however, both the companies and their products are relatively short lived for many reasons. For example, current social robots succeed in interacting with humans only within controlled environments, such as research labs, and for short time periods since longer interactions tend to provoke user disengagement. We interviewed 13 roboticists from robot manufacturing companies and research labs to delve deeper into the design process for social robots and unearth the many challenges robot creators face. Our research questions were: 1) What are the different design processes for creating social robots? 2) How are users involved in the design of social robots? 3) How are teams of robot creators constituted? Our qualitative investigation showed that varied design practices are applied when creating social robots but no consensus exists about an optimal or standard one. Results revealed that users have different degrees of involvement in the robot creation process, from no involvement to being a central part of robot development. Results also uncovered the need for multidisciplinary and international teams to work together to create robots. Drawing upon these insights, we identified implications for the field of Human-Robot Interaction that can shape the creation of best practices for social robot design.

## 1 Introduction

How does a designer start to create a social robot? Our work lifts the curtain on a topic thus far unexplored: how robot creators, from industry to research labs, design and fabricate social robots. We shed light on current design practices for social robots and derive specific implications for the emerging field of human-robot interaction (HRI). By identifying limitations and challenges inherent in social robot design, we intend to inspire use of best practices for their creation, helping researchers and commercial designers build higher quality products that are better suited for consumer markets. Our ultimate goal is to inspire robot creators to build social robots that are closely aligned with humans needs and values within the socio-technological society in which we live.

The term *social robot* has been used to define ‘socially interactive robots’ (Fong et al., 2003) that have one or more of the following competencies: the ability to communicate, express affective behaviors and/or perceive human emotions, have personality or character, model social aspects of humans, learn and/or develop social skills, and establish and maintain social relationships (Shibata, 2004; Yanco and Drury, 2004; Dautenhahn, 2007). Therefore, designing social robots requires a combined understanding and knowledge integration about human behavior and intelligence, as well as a diverse set of technical skills, e.g., in computation and fabrication (Baraka et al., 2020). This makes social robot design intrinsically interdisciplinary compared to the design of other artifacts or technologies.

In this paper, we provide recommendations for future robot creators to inspire the design of successful social robots. We conducted in-depth, qualitative interviews with expert robot creators from companies and research labs who are directly involved in the design, development, and testing of social robots. During our interviews, they disclosed their design process, the extent to which end-users were involved (if at all), and how their teams were composed.

## 2 Related Work

### 2.1 Uniqueness of Social Robots

Unlike industrial robots, which have been on the market for some time, social robots are a newly emerging technology just now appearing in our stores. In this work, we deliberately chose to interview robot creators and analyze their design processes. Previous research examined how digital fabrication tools, such as 3D printers, laser cutters, and CNC routers, are fabricated by interviewing professionals that utilize these tools (Yildirim et al., 2020); it highlighted practices concerning the use of digital fabrication tools, specifically focusing on machine awareness, autonomy, and user agency. While these findings are relevant to the field of robotics—especially because the initial stages of creating a robot involve using digital fabrication tools to build prototypes—it does not explore the design process for social robots, which we address in this paper.

Sanneman et al. (2021) described the processes and challenges that companies follow when working with new technologies such as robotics and the Internet of Things (Sanneman et al., 2021). Insights from interviews with key players in the industrial robotics ecosystem contribute to research directions for the field of industrial robotics. While this work is relevant for the field of HRI since many components of social robots are shared with industrial robots—including vision, perception, and control—our work focuses more deeply on the challenges inherent to designing interactive robots that communicate with people, also fertile ground for investigation.

Previous work on robot teams explored the attitudes of frontline employees who use industrial robots every day (Sauppé and Mutlu, 2015; Elprama et al., 2017; Wurhofer et al., 2018; Welfare et al., 2019). Additionally, an extensive ethnographic investigation studied anthropomorphism in teams that work with robots (Chun and Knight, 2020). While these studies focus on the team that directly works side-by-side with robots, our work focuses on the experience of teams that design and build new robots.

By acknowledging the uniqueness of social robot design, Axelsson et al. introduced a framework for participatory design practices for social robots (Axelsson et al., 2021). This framework provides templates and guidelines to promote collaboration between multidisciplinary teams when creating social robots. This approach relates to ours; however, the authors neither explored the inclusion of users in the process of social robot design nor accounted for the benefits and shortcomings of different Human-Centered Design (HCD) practices applied to this problem, which we uncover in this paper.

### 2.2 Product Design and Development

The product development cycle is characterized by multiples theories and practices (Moni et al., 2020). During our research, we interviewed robot creators about the life cycle of creating a social robot. We highlight below some of the more influential practices in product design and development to better contextualize this research. Note that benefits and costs apply to all approaches, which generally work in combination rather than individually.

A *linear design process* is primarily used to manage risk when conceptualizing a product. In this practice, each phase must be fully completed before proceeding to the next, letting designers catch errors when they are least expensive and time-consuming to fix. The linear method is straightforward but requires discipline to be effective. However, during the design process, it is essential to realize that most use scenarios will require flexibility and the ability to react to new information and circumstances, challenging considerations in this linear practice (Bocken et al., 2016).

In contrast, *user-centered design (UCD)* is an iterative design process in which designers focus on users and their needs in each phase. User-Centered Design (UCD) teams involve users *via* a variety of research and design techniques to create highly useful and accessible products. There exists an explicit understanding of the users, tasks, and use environments: the aim of the process is to capture and address the whole user experience. Therefore, the design team includes professionals drawn from multiple disciplines, and experts may conduct evaluations of the produced designs using design guidelines and criteria (Still and Crane, 2017). This work uses the term *human-centered design* (HCD) to address inclusion of user emotional or psychological preferences (Gasson, 2003). Examples of HCD practices include the body of work by Don Norman (Norman, 2013) and the 7 Principles of universal Design (Story, 2001).

Finally, when using *design heuristics*, domain experts and users can assess product usability. This rapid design evaluation calls upon domain experts to go through checklists aimed at assessing the system’s (or the robot’s) ‘heuristics’ to guide future improvements (Jiménez et al., 2012). Such methods are common in the design of a variety of products and also contribute to the design of social robots.

### 2.3 Social Robotics Market

According to a 2020 market analysis by BCC Research, social robots are a rapidly growing market, with a compound annual growth rate of around 15% (LLC, 2020). According to Statista ^1^, in 2018 social and entertainment robot sales reached 2.68 million units worldwide. By 2025, that number is forecast to double to 5.51 million units. Thus, we see an emerging recognition of the potential of this field, especially for use in healthcare, education, and entertainment.

Despite this growth, many robot companies have failed in the market. In 2019, the *Robot Report* ^2^ released information about social robot companies that ceased production; it noted that many initially thriving companies failed to succeed over the longer run. Our work aims to be a conversation-starter on the topic of social robot failure versus success by lifting the curtain of a topic that is often discussed but little studied in the HRI community: the inner workings of social robot design.

## 3 Methods

After having developed our own social robot prototype (Björling and Rose, 2019) for the Ecological Momentary Assessment Robot (EMAR) project, we wanted to learn from other researchers and designers of scalable robots currently on the market to assess how best to scale our prototype. Our research questions were:

• What are the different design processes for creating a social robots?
• Are users involved in the design of social robots, and how?
• Who participates on the teams that develop social robots?

### 3.1 Collective Case Study

We conducted an *interview-based qualitative case study*, an established research design method, where we consulted a variety of stakeholders involved in the creation of social robots. Case studies enable in-depth appreciation and multi-faced exploration of complex issues or phenomena of interest (Crowe et al., 2011); their value in research lies in their ability to explain, describe, or explore events (Yin, 2017). Unlike experimental designs, which focus on testing a specific hypothesis by deliberately manipulating interventions or conditions, the case study approach captures information of a more explanatory nature by focusing on ‘how,’ ‘what,’ and ‘why’ questions.

Our case study focused on identifying and comprehensively describing how social robots are created. Specifically, we applied a *collective case study approach*, which simultaneously explores multiple cases in an attempt to generate broader appreciation of particular issues (Stake, 1995). Thus, our collective case study included employees from multiple social robot companies as well as professors from university labs that build social robots.

### 3.2 Ethics and Permissions

This study was reviewed by and received Institutional Review Board approval from the University of Washington, Seattle, WA, United States. Participants verbally consented to be recorded. To protect participants, all information that could potentially lead to identification of individuals was removed, and transcripts were anonymized.

### 3.3 Sampling and Recruitment

We used the purposive sampling technique to recruit subjects for this study. *Purposive sampling* is a form of non-probability sampling in which researchers deliberately choose participants due to their unique qualities (Tongco, 2007); it is one of the most effective techniques to study a specific domain with knowledgeable experts, which is the case for our study. Participants were identified by 1) drawing on the extended network of authors of this paper, and 2) applying the inclusion criteria that recruits required hands-on experience in creating social robots. We specifically focused on subjects who worked with social, interactive robots rather than industrial ones: these two markets require different knowledge and experience, are associated with different application scenarios, target different users/consumers, and exhibit diverse market maturity, with industrial robots being used in the marketplace for far longer.

**FIGURE 1:**
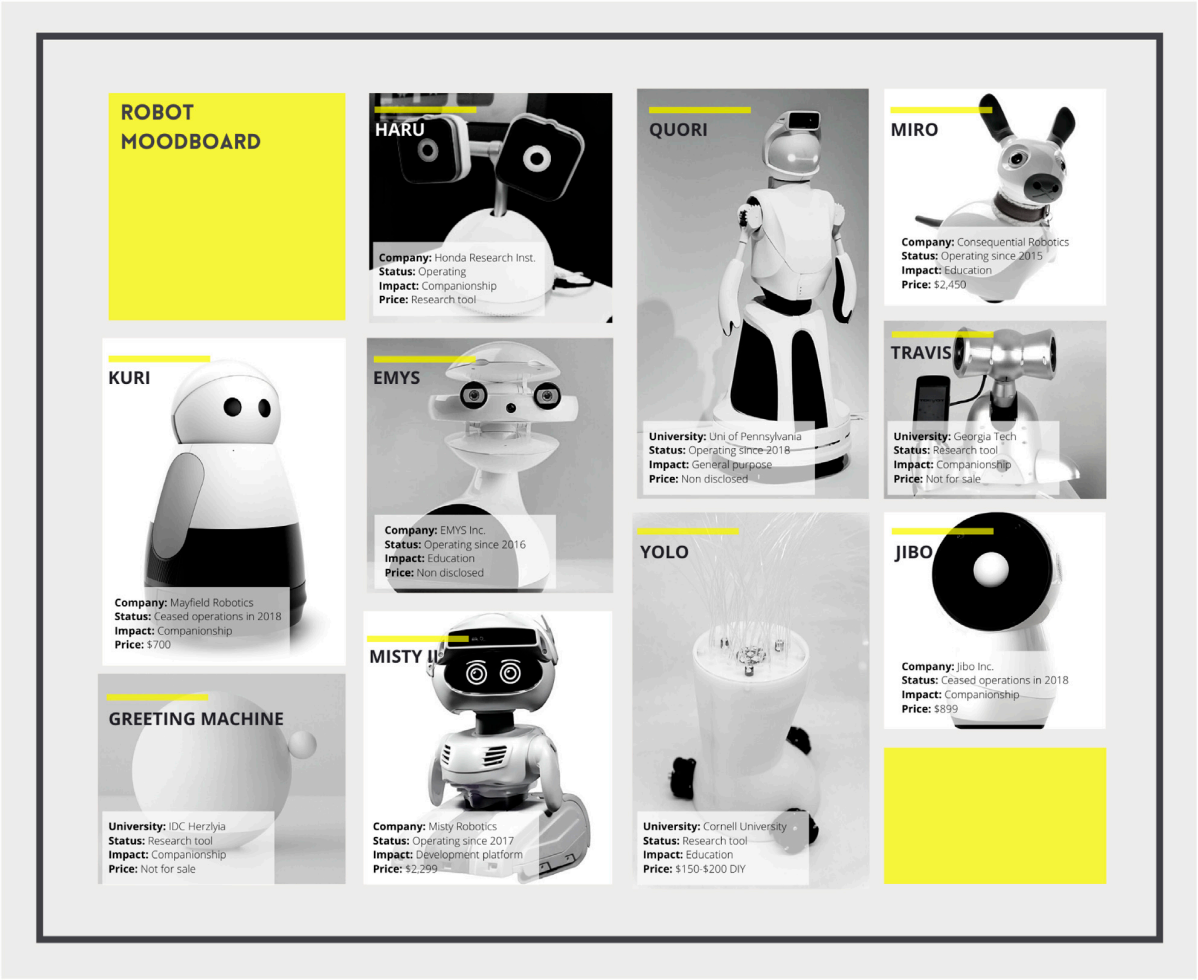
Moodboard of robots included in this study, including details of the robot’s founding company or university, business status, area of impact, and selling price. Prices may vary from those noted here.

We identified 18 subjects who had created one of more robots in the context of a research lab or in industry. We sampled purposefully for maximum variability, ensuring representation from a range of countries, professional backgrounds (including engineers, designers, artists, system developers, academics, and visionaries/futurists), and types of robots (Patton, 2005). The initial recruitment email included an invitation to participate in an interview about their role in the design of a social robot along with sample questions from the interview template. A total of 13 subjects expressed an interest; the follow-up email contained the interview schedule; refer to the demographic description in Table 1 and the robots build by these creators in Figure 1. The remaining 5 subjects who did not enroll in this study mentioned either their concern about discussing the topic given their non-disclosure agreement (NDAs) (2 subjects) or simply failed to reply to our email solicitation (3 subjects). Industry representatives were particularly uncomfortable with sharing potentially sensitive commercial information. We stopped recruiting additional participants when we reached thematic saturation, which occurs when no new themes emerge during analysis (Guest et al., 2006).

**TABLE 1:** Demographic description of study subjects. All subjects are robot creators who designed and built one or more robots in a research lab or company.

Subject ID	Gender	Country	Role
1	Male	Poland	Co-Founder and Electrical Engineer
2	Male	Israel	Industrial Designer & Hardware Engineer
3	Female	Australia	Designer and Animator
4	Male	US	Robot Animator
5	Male	US	Software Engineer
6	Male	US	Software Engineer
7	Female	US	Chief Operating Officer (COO)
8	Female	US	Software Engineer
9	Male	United Kingdom	Co-Founder and Designer
10	Male	US	Senior Engineer
11	Male	US	Founder and Principal Investigator
12	Male	US	Researcher
13	Female	Portugal and US	Researcher

### 3.4 Data Collection

Among the wide range of qualitative methods, we specifically chose to conduct interviews, which enable flexibility during data collection while remaining grounded in a particular framework (Gill et al., 2008). Interviews were conducted over Zoom ^3^, digitally recorded, and transcribed. They ranged from 30, − ,90 min depending on the subject’s availability and how in-depth the interview went. We explored the most promising areas, including the development process of robots, user or customer involvement in the robot creation, and team composition of the robot builders. A sample interview guide is shown in the box below. While several pre-defined questions employed a blend of closed- and open-ended formats, we often accompanied these with follow-up ‘why’ or ‘how’ questions. This enabled flexibility to explore novel topics raised by the subjects while having a template that guided the discussion (Dearnley, 2005; Newcomer et al., 2015).

Interview Sample. Questions were distributed according to the themes of interest in this study.

• *What was the workflow that you followed for the development of the robot?* (robot development process)
• *What types and how many robot prototypes did you explore?* (robot development process)
• *How different were the prototypes compared to the last version of the robot?* (robot development process)
• *Were users or consumers involved at any stage of the robot creation?* (user involvement)
• *What type of data was collected, and how did it inform the development of the robot?* (user involvement)
• *What were the backgrounds of the team that created the robot?* (team composition)
• *How was the division of labor distributed across the team?* (team composition)
• *With this particular robot today, what are the current pain points?* (lessons learned)
• *What would you do differently if you were to do this again from the beginning?* (lessons learned)

### 3.5 Data Analysis

We identified different design processes for robot creation, several degrees of user/customer involvement in the creation, and different approaches to team composition of robot builders. We anchored our data analysis in qualitative research methods, suitable methods for exploratory studies such as ours that support inductive practices; these methods can lead to prominent emerging themes without existing prior hypotheses (Sofaer, 1999). While quantitative research could potentially be useful, a growing consensus indicates that they are ideal to justify research findings or differences across samples (Park and Park, 2016). In contrast, qualitative research is concerned with aspects of reality that cannot be easily quantified, focusing on the understanding and explanation of a phenomenon and thus deepening our comprehension (Queirós et al., 2017). This was compatible with the goal of this study, which aimed to deepen the understanding of the processes and approaches used to design social robots.

Transcribed interviews were uploaded to Miro ^4^, an online collaborative whiteboard suitable for research analysis that enables visual organization of data and exploration of prominent themes. Three researchers were involved in collaborative coding of the data. Two researchers independently organized the interview materials into emerging themes. To ensure consistency across coders, calibration exercises were performed until stability was reached (Krippendorff, 2009). After coding 30% of the data, the two coders met to resolve discrepancies (Campbell et al., 2013); they compared their coding schemes to ascertain concordances (i.e., alignment in definitions, language, and coding logic). When discrepancies arose, a ‘negotiation agreement’ was used, whereby they verbally discussed differences with a mutual effort to reconcile disagreements and divergence (Hoyle et al., 2002; Garrison et al., 2006). The third coder joined the discussion when 50 and 100% of the data was coded to help disambiguate negotiations.

We approached the analysis with an initial coding framework based on our research questions to provide an initial structure to our findings. We used an *affinity diagram* approach to code and organize the data (originally called the KJ method) (Kawakita, 1991). Affinity diagramming is a technique used to externalize, make sense of, and organize large amounts of unstructured, far-ranging, and possibly dissimilar qualitative data (Hartson and Pyla, 2012). Data collected in our interviews occasionally ventured in directions that differed from our primary research focus due to the nature of open-ended questions and semi-structured interviews. While these extraneous data were interesting, if it was not relevant to our research questions, it was not included in our results.

## 4 Results

We now present our findings on several robot development processes, the different degrees of user/customer involvement, and the team composition of robot builders.

### 4.1 Robot Design Process

**FIGURE 2:**
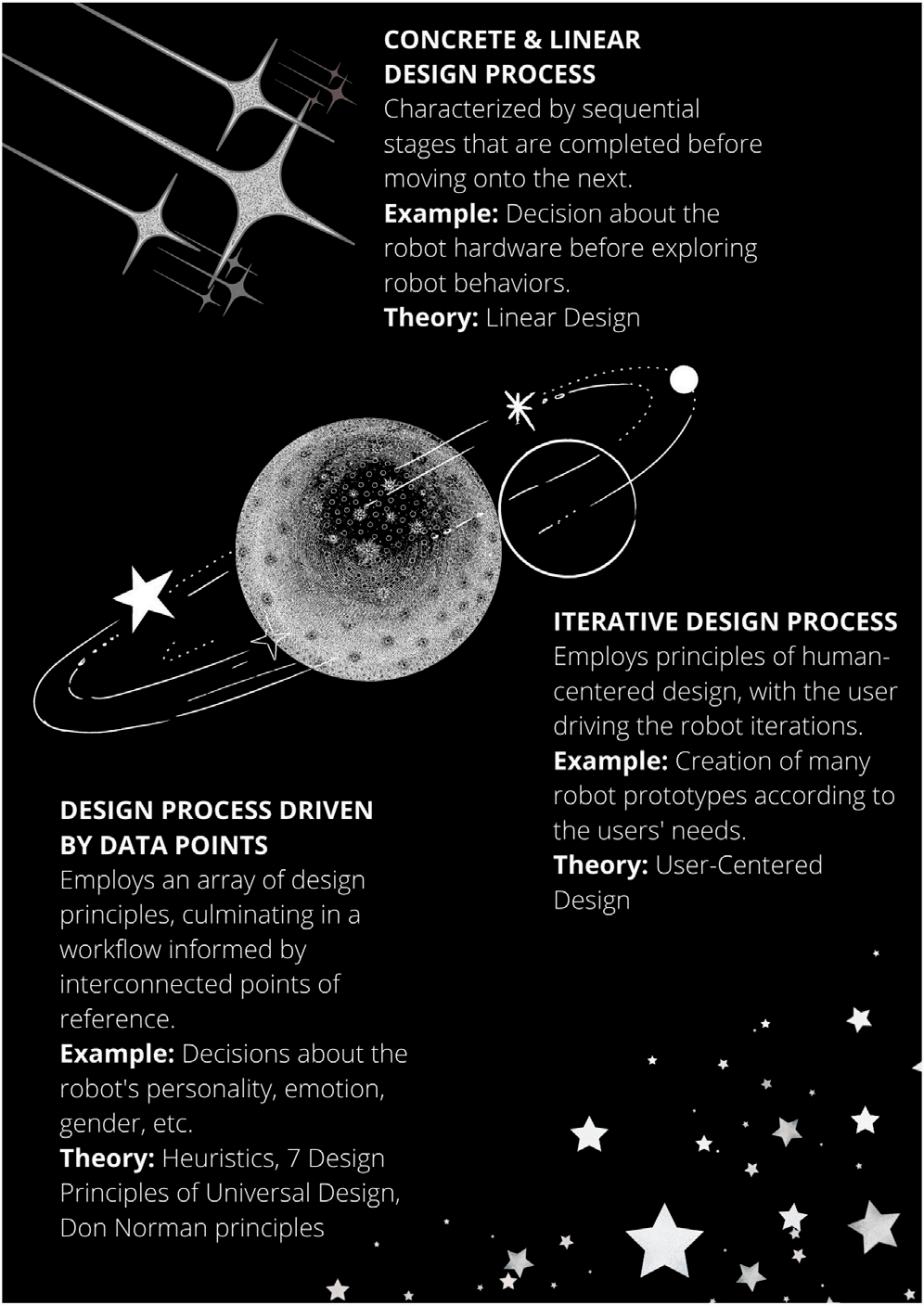
Design process of social robots.

Three categories of design processes can be derived from our data: 1) iterative, 2) linear, 3) and data-point-driven (see Figure 2). We extracted the most salient details of each development process to provide a deeper understanding of the various workflows. Further, these categories are not mutually exclusive, and certain aspects overlap with others, meaning that the same robot can be mapped to more than one design process. We do not aim to compare the effectiveness of one design process to another or to state preferences; rather, we provide a comprehensive illustration of the current way robots are being designed, which has inherent value.

#### 4.1.1 Concrete and Linear Stages of Social Robot Design

A *linear robot design process* refers to concrete and sequential stages of a workflow underlying the robot’s creation. Drawing from key tenets of linear processes in design, each phase in the development life cycle should be completed before moving to the next. An example was described by Subject 10 in five well-defined stages: 1) hardware exploration, which consists of creating initial prototypes and sketches of physical features for the robot, 2) design investigations, which involves experimenting with simple robot behaviors within interaction scenarios, 3) expressivity implementation, which consists of creating 3D printed mock-ups to test and refine degrees of freedom, 4) interaction design, which uses a puppeteer and stop motion artists to test which degrees of freedom are actually needed in the robot and which can be removed, and 5) negotiation, where the team navigates conflicting aspects of the design, striving to balance market viability and mechanical feasibility. A description of five of the created robot prototypes follows:

*“Our first [prototype] was just a base platform. The question was: can we make it work? Our second [prototype] was this concept we liked, but we couldn’t actually get it to be expressive enough. The third one has the degrees of freedom in a different place. The fourth one was pretty much the robot that you probably see now. The fifth one has some tweaks here and there, and, beyond that, there were other little tweaks.”* (Subject 10, Male)

These sequential stages were described as one leading to the next, with the ultimate design decisions driven by *“this tension between how much expressibility we want the robot to have, how much it is going to cost us, how much can we sell it for, and how do we want people to interact with it.”* (Subject 10, Male).

The development process described by Subject 6 included not only an iterative process but also linear stages. The overall process was conceptualized as a series of phases, *“We did go through several phases of the robot [development]. Each one of those phases lasted a few months, maybe* 4 − 5* months each”* (Subject 6, Male). The first stage was described as *“bare bones utilitarian”*, which consisted of a prototype with finished electronic components and an unfinished exterior. This featureless version was used to evaluate the electronic components, tasks, and flows of the robot. The following three versions were assigned labels by the manufacturing team and used to perform lifecycle and long-term testing and to make various other refinements. Each phase of building was completed before moving on to the next.

Another example of a design process with clear sequential phases consists of phases that can be charted along a timeline of 4 years:

*“In year 1, lots of prototyping, need-finding, taking prototypes out to get customer feedback, working with the industrial designers, working with mechanical designers, prototyping navigation software and drive trains and animation for the degrees of freedom and depth sensors. In the second year we hired our VPs [vice-presidents], developed alpha versions of the robot, depth sensor, at the end of the year prepped to launch at CES* ^5^ *with our painted prototypes. In the third year we launched at CES, did design for manufacturing, and at the very end of the year, shipped our first small batch of robots out to pre-order customers. In year 4 we scaled up production, got shut down just as we had our full-speed manufacturing line set up to turn out thousands of Kuris every month.”* (Subjects 7 and 8, both Female)

#### 4.1.2 Iterative Process of Social Robots Design

The*iterative development process* enables continuous improvements of the robotic system and a deeper understanding of users and their needs. A subject described this iterative design process in the following way:

*“We went through a lot of iterations of sketching and then some low fidelity prototyping with cardboard. And the process used a lot of increasingly high fidelity prototypes with constant feedback, preferably from users. It’s a kind of classic user-centric design process in many ways.”* (Subject 2, Male)

Another subject described a similar iterative process. The process began with aiming for simplicity and speed because *“the first prototype is going to suck anyway and you will miss the target”* (Subject 1, Male). In this case, each iteration brought with it more learning, failure was acknowledged to be part of the development cycle, and the finished robot design resulted from numerous prototypes:

*“[the development process of the robot] was very, very, very iterative. That’s one of the things that, you know, when you start building a robot, you think you will build one, and NO. You will build many, many, and many more than what you think you will.”* (Subject 2, Male).

He added:

*“You do the first [prototype] and it doesn’t work or works badly. Then, you learn and make it again, and it gets better. By the time you make it ten times, it’s pretty good.”* (Subject 2, Male)

The underlying approach of the iterative development reflects the idea that iteration strengthens one’s knowledge and culminates in *“a good enough robot that works”* (Subject 2, Male). Furthermore, this approach addresses user needs and identifies pain points: *“You will want to build something that is meaningful, and that means building something that people actually want and that can solve a problem in the world.”* (Subject 2, Male).

The users of social robots, though central, are not the only references that developers rely on. For example, internal team feedback was considered crucial since the team is also composed of expert roboticists that can contribute to the problem. The team would build mini prototypes of a certain robot feature or interaction case, and the internal design teams would review them and offer suggestions. They would then incorporate feedback into the next prototype and repeat the process, as noted by Subject 6:

*“And we’d iterate back and forth on the interaction design of the skill, and then we’d go off and sort of build another version and show it to them [the team] on an average of every 3 weeks or every month kind of cadence.”* (Subject 6, Male).

The iterations were driven by the goal of building a robot with minimal areas of weakness: *“at least five iterations [of the robot], and then one big robustness redesign where we’re trying to fix all the things that just don’t work well”* (Subject 12, Male). The iterations can be driven by a specific feature to improve in the robot. For instance, while a robot can have multiple iterations, the main motivation for them was to test different designs and physical materials, not to make users the focal point of the orbit: *“We have possibly 10 versions of the robot before we have the one that we have now and, you know, we have 10 iterations of those, like different mechanical designs, different materials, just small things.”* (Subject 12, Male).

Subjects 1, 2, 6, and 12 took iterative approaches when building robots; however, Subjects 6 and 12 described workflows different from those of Subjects 1 and 2 since their orbits were more focused on gathering feedback from internal teams or improving specific features, rather than being driven by user feedback. Thus, an iterative design process does not necessarily mean it is user-centric, but rather that different expertise can be considered in the iterative process (such as the internal expertise of the team).

#### 4.1.3 Data-Point Driven Robot Design

In a data-point-driven robot design process, the workflow is informed by points of reference, such as prior knowledge or accumulated observations. Instead of concrete stages, data points do not adhere to a specific timeline; rather, they serve as important references throughout a whole development process. Various theories and principles are associated with this method, such as Don Norman’s definition of affordances, the Seven Principles of universal Design, and Jakob Nielsen’s Ten Usability Heuristics, showing how this design process pulls data points from different disciplines, frameworks, and sources of knowledge.

An example was described by Subject 3, who drew on various data points when designing the robot, including her own background in design and animation, affinity for understanding the human experience, and prior research in the field of robotics. For Subject 3, the philosophy around developing social robots is that they should spark feelings and emotions in the people who use them. She mentions the importance of having companies that support this approach: *“They’re brave to design a robot that brings joy”* (Subject 3, Female). Along with this philosophy was the importance of designing communication for joyful interactions mapped onto the robots’ specific features and components. Subject 3 highlighted the importance of communication beyond speaking, *“What do we do when we’re being social? We don’t just talk. What’s the content of that talk, and what are the things we do?”* (Subject 3, Female). Elaborating on this idea, creating a social robot that brings joy is exemplified in her knowledge of human behavior, which served as data points that drove the aesthetic and gesture selection design of the robot:

*“Many of the robots in the marketplace are designed for movement, which is just motion. And not designed for gesture, which is emotion.”* (Subject 3, Female)

Subject 3 explained how prior experience informed her approach to aesthetic robot design as follows:

*“I come from an animation and design background. In my design, I always worked to aesthetic principles, which are super important to robotics. Aesthetics are important to understanding a robot’s purpose, what it should be used for, and how somebody relates to a robot.”* (Subject 3, Female).

An additional reference point elaborated upon by Subject 3 was the key insights from surveys and interviews with the target audience that would use the robot being created. A major finding dealt with people’s expectations and comfort with social robots:

*“In surveys I’ve done, there’s been a rebellion against perfection in a robot, and rebellion against the robot being a know-all, and also a rebellion against humanoid robots. Also, that they [the target audience] didn’t want robots to be gendered.”* (Subject 3, Female)

Overall, these data points ‘interacted’ with one another to form the larger approach of developing a robot. Subject 3’s philosophy, background, and research insights were not disparate elements of robot creation; they were instead interwoven data points that the team drew upon when designing the robot.

Another example came from Subject 9. For instance, research informed the placement and design of the robot’s eyes:

*“Stereo vision is very important. A rabbit has eyes on the side for predators. The same for cows and horses. Instead, cats and lions look straight ahead. So, the peripheral placement is less threatening and is cuter. This part is informed by research, and the robot was made with peripheral eyes. However, we placed the cameras to see straight ahead and not lose robot’s functionality.”* (Subject 9, Male)

Insights derived from researching animal features revealed the importance behind eye placement. Since this robot was intended to be *“a cute animal”*, mainly to have impact on the educational sector, its eyes were positioned on the side of the face to be perceived as approachable and friendly. Subject 9 also relied on his basic understanding of human behavior as a point of reference in the design process. The decision to model the robot after a pet was driven by his perspective that humans view their pets as companions:

*“Everyone talks to their pets. Find me someone that does not talk to a pet. But does the pet understand?”* (Subject 9, Male)

An important data point is the background and experiences of robot builders. Arguably, each subject drew upon unique experiences and knowledge when designing the robots. Subjects 10 and 11 explicitly mentioned expertise as a driving force. For example, *“when the focus was hardware, the improvements were made based on failures and expertise”* (Subject 11, Male). Similarly, relying on their background in robotics and HRI when faced with design decisions was important: *“A lot of [the development process of a robot] was all about just trying to take the lessons learned. I’ve been in HRI for how many years, and it is like, hey, this is what people have shown so far. How do we apply these principles?”* (Subject 10, Male). Thus, a main data point is the team’s expertise and unique background, which can inform the process of robot development and the underlying decisions made.

### 4.2 User Involvement in Robot Design

**FIGURE 3:**
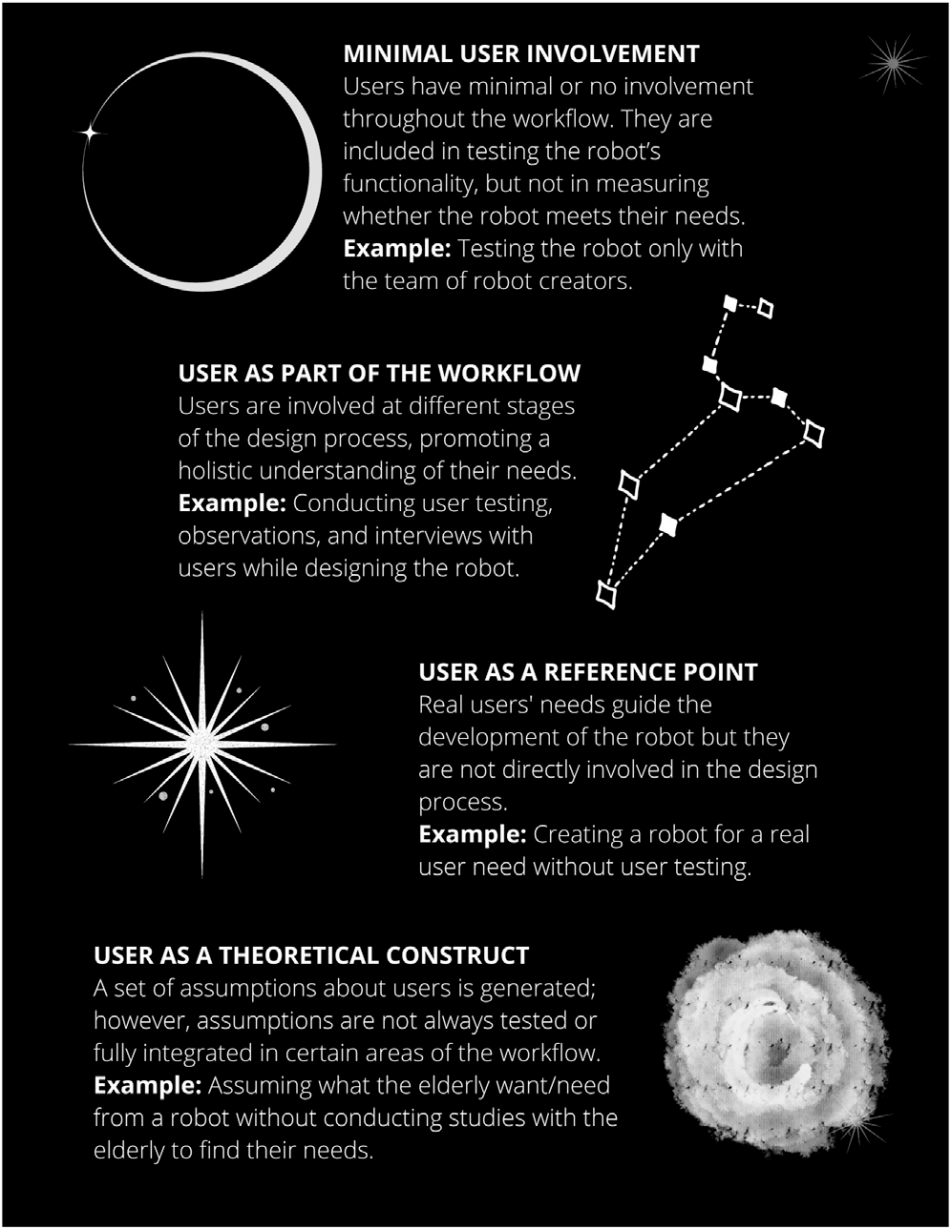
Different degrees of user or customer involvement in the development of a social robots.

We identified three degrees of user or customer involvement in social robot development (see Figure 3). We note that in some stages of the robot’s development, users can be involved in more than one way. Further, it is not our intention to map specific robots to specific degrees of user involvement.

#### 4.2.1 Minimal User Involvement

This category includes robot development workflows that consider minimal input from users during robot design and testing. Here, users lack a concrete presence/identity and are brought in to test the robot’s functionality, not to measure whether the robot meets deployment needs (e.g., cost, engagement in the interaction). In this case, users’ involvement is limited or nonexistent throughout robot development. Subject 10 mentioned that the team did not have a specific target user in mind throughout design and testing. This can be seen in the minimal user involvement, especially in the testing and evaluation stage:

*“Mostly, what we were able to do was show to other people in the company who weren’t working directly on this robot…Sort of like grabbing another person in your lab and being like, ’Hey, look at this.’ It wasn’t as formal as a user study.”* (Subject 10, Male)

In this case, the robot was tested within the team instead of with actual users or customers who would be buying and using it. In terms of design, most of the larger decisions were made by the team without direct user input. For instance, the decision to include prominent feminine features in the robot was made solely by the team, none of whom were women. This decision was a topic of contention among customers and stakeholders:

*“We got push-back because the silhouette [of the robot] has a very thin waist and a prominent chest area. People sort of mentioned that was perhaps feeding the stereotype. There’s this whole thing about gendered robots and gender perceptions, and we probably could’ve done a better job with that aspect.”* (Subject 10, Male)

As such, not involving the users or customers who represent specific demographics in the design stage negatively affected the outcome and resulted in perceptions of stereotypes perpetuation that were neither initially intended nor considered. This was identified as a pain point and a reflection for different future decisions when creating robots: *“I would probably fight harder to bring other voices to the table. I think there’s a strong view of ‘Oh, we know what we want to build,’ and less input from potential customers.”* (Subject 10, Male). This demonstrates the importance of defining the target user to guide the direction of robot design, gathering insights on their needs, and including them in design evaluation to ensure their needs are met.

Another example of a low-level of user involvement in development is stated by Subject 6: *“I don’t think it was a super user-driven design. We didn’t have a ton of users.”* (Subject 6, Male). Instead, the design was driven by *“the simplest possible mechanism that could still give us a wide range of expression and expressive motion”* (Subject 6, Male). Here, the workflow behind this robot seemed to prioritize optimization over user involvement.

#### 4.2.2 User as Part of the Workflow

Through the lenses of HCD, users are central parts of the design flow when they are included in different parts of the robot development process. Knowledge gathered from the users at different design stages enables a holistic understanding of their needs, much like an outline or pattern of what would be desired in a robot. This informs the research with a specific population of user views to reflect in the design requirements (Cooley, 1999; Buchanan, 2001). For instance, Subject 3 uncovered many key insights from user input when designing the robot, which helped her understand what users want in a robot and why:

*“I did a survey on the robot with the target age group. And what was interesting about that age group is that they didn’t want a robot to share. They wanted a robot for themselves. They wanted that robot in their room. They wanted it because they spent a lot of time in their rooms. They wanted a study buddy but also wanted something that I suppose wasn’t threatening. It was like social media and everything, but something that was kind of like their own friend, that was just theirs.”* (Subject 3, Female)

User insight drove design decisions concerning how to convey the robot’s purpose, character, and story. Instead of asking customers for their desired features, the team let users drive the design by investigating the underlying feelings behind companionship and how they might interact with a robot. From there, the team created physical representations of the user’s feelings. For instance, the need for a non-threatening robot that could act as a study buddy or friend informed the design of communicative features, non-humanoid design, and genderless identity.

Subject 2 took a similar approach in centering the user in the workflow. One process involved designing a companion robot for the elderly population to help them cope with loneliness, as expressed below:

*“We did a lot of interviews with the elderly where we showed them different types of robots, and we did thematic coding of what they think of these different robots. We then made guidelines for designing a complex social robot for the elderly, and a few things came up in the end that we used as guidelines for the design of other robots.”* (Subject 2, Male)

Through interviews with the target demographic, they were able to drive the design of the robot through guiding design requirements. From the user’s input, they *“designed a social robot, which has social features aiming at giving people the feeling of being seen.”* (Subject 2, Male), which was one of the most salient needs amongst the elderly population they interviewed. Furthermore, the users were continually involved throughout the other stages of design. In addition to using interviews, robot developers applied other methods to understand the users and gather their input. For instance, they *“took videos of different robots that represent different kinds of robots. We showed them a few different robots and got information from them.”* (Subject 2, Male).

Different methods were used according to the competencies of different users: *“For 2 years, we observed how children play with the robot prototypes that we gave them. They were always part of the process; it just didn’t work asking them what the liked or not, we just need to sit still and observe. This would tell us what needed improvement.”* (Subject 13, Female). In this way, the user was a central part of the workflow and drove robot design. The different inputs gathered at different design stages enabled a constellation of knowledge about the users’ needs, desires, and wishes for the robot.

#### 4.2.3 User as a Reference Point

Users can also act as reference points that drive the development of a robot. In this case, the design process consists of a fine balance between user input, designer’s decisions, and business or time constraints. For instance, Subject 1 elaborated on the intricate dance of including customers in the development of the product while maintaining the designer’s vision: *“The idea that you have is still important, and it is a very, very fine balance because you have to make sure you are building something for the users, but it is also very easy to ask the wrong questions here.”* (Subject 1, Male).

After asking users what they want, robot builders can be inundated with complex features ideas that increase robot costs. There might be reluctance to let users drive the design process since the process can become subject to the ‘feature creep’ phenomenon [(Thompson and Norton, 2011)]:

*There is a name in the start-up world, which is ’feature creep,’ where basically you keep on adding, and adding, and adding, and adding features to your product because, well, your customers are asking. It’s actually a huge risk, and it kills companies because it takes time to develop features, and it’s very, very costly to develop something that’s wrong because it’s something that the users say they want or need, but it’s not something that they would need so much that they would pay for your product.”* (Subject 1, Male)

Feature creep introduces the dilemma that exists when asking for user input. On one hand, it is important to ask for and integrate user feedback in order to meet their needs. On the other hand, including user input runs the risk of adding features to the point of driving up costs, developing something that is not marketable, and creating an overly complex product. Instead, there are ongoing negotiations about finding balance between user involvement and the designer’s visions for the robot. One way to achieve this balance can be to include users in overcoming major pain points of the robot’s design:

*“The way to think about that is to not build something that does everything, but to have the design process set up so that you actually build for one thing that is very, very, very specific and that solves a very big pain of your customers or users.”* (Subject 1, Male)

They refer to this approach as *“solution viability”*, which is related to the idea of *“building something that is so good and solves such a big problem that people are actually willing to give you money for it, regardless of whether you ask them for this money or not.”* (Subject 1, Male).

#### 4.2.4 User as a Theoretical Construct

The user can also be included as a theoretical construct or as a simulation. For the former, a set of assumptions about users is derived from the experience or specific pain points of the team who created the robot; however, these assumptions are not always tested and might lead to biased decisions during robot development. For example, among the target users for MiRo are children who are *not* interested in coding, despite the robot being developed to teach programming skills to children:

*“A lot of kids that put aside coding in school are enjoying teaching a cute animal, which is what it [the robot] is intended to be. This is because we want to bring creative kids to coding and interacting with the robot, because a lot of kids are not into coding.”* (Subject 9, Male)

This example shows how the robot’s target user differed from the theoretical construct held by the team based on their previous experiences. Subject 9 elaborates on this: *“There was also a team that when designing [the robot] expected it only to be used for university students and not kids or the elderly, so they were not included at the time in the design.”* Although the subset of target users was considered during certain stages of design, they were not fully integrated in all areas of the workflow. In this case, assumptions were made about children’s low interest in learning to program that were not always investigated. Instead, this user demographic existed in their design but served as a theoretical construct with limited actual involvement. While this resulted in positive outcomes for this particular robot, *“Unintentionally, it [the robot] has been more successful than what we thought it would be.”* (Subject 9, Male), this is not true for many robot companies that eventually cease operations.

Users can also be simulated using algorithms. In this case, instead of testing the robot with real users, the team can perform a series of virtual simulations to assess how a robot would behave in an interaction scenario among people. Another situation can take place when users are asked to provide feedback of a virtual, simulated robot. While the feedback from users can be valuable, their experience of interacting with a virtual robot can significantly differ than their experience interacting with a physical robot, which can bias the design and development process in ways that are not optimal for user adoption. For example, *“We developed a questionnaire for HRI researchers where they were asked what they wanted in terms of degrees of freedom for the robot.”* (Subject 11, Male). However, the team quickly realized that users wanted more than what was feasible to achieve in a physical robot (compared to the virtual robot shown in the questionnaire). Thus, we observe the necessity of testing a physical robot with real people during robot development, where substantial changes can be made, if necessary, to avoid biases about users needs.

### 4.3 Team Composition When Designing Social Robots

Several main topics emerged when discussing the composition of teams that create robots: 1) all interviewed subjects belonged to *interdisciplinary teams*, 2) the majority of teams used *outsourcing* for special skill acquisition, 3) most teams relied on *international sites* to manufacture scalable robots. This section describes these topics and discusses the human dynamics underlying the challenges and success of these teams.

#### 4.3.1 Interdisciplinary Teams

To create a social robot is to create an artificial being. Therefore, the design, development, and testing of social robots calls for *interdisciplinary team composition*. Reviewing our subjects’ backgrounds (Table 1), we see that teams are generally composed of mechanical and electrical engineers, computer scientists, psychologists, and artists.

When referring to how their team is composed, it was mentioned: *“We had the two founders and CEO, so they have an electrical engineering background and a design mechanical engineering background. We had a mechanical engineer, a very serious developer (really top notch), a second electrical engineer, and then me. And I think we had a second 3D designer.”* (Subject 10, Male). Another subject highlighted the richness of social robot design when knowledge from different fields is incorporated:

*“I come from an animation and design background. I always worked to aesthetic principles, which is super important to robotics.”* (Subject 3, Female)

This idea was further reinforced:

*“The team brought in different insights. I brought the HRI part with the human scenarios, then there was the hardware of how to actually build it, and the designers were about how can we shape it. They knew what really looks good.”* (Subject 10, Male)

All interviewees mentioned they had interdisciplinary teams and acknowledged the complexity of creating a social robot. The main insight here is that *interdisciplinary teams have the required knowledge to create social robots.*

#### 4.3.2 Outsourcing Special Skills

Despite the necessity of working with interdisciplinary team members, not all team members are needed at all stages of robot development, and some roles are outsourced. This brings the advantage of decreasing the complexity of the ‘core team’ of robot builders and of making the product scalable and successful: *“The most robust robot that we built was with a collaborator, who was a mechanical engineering consultant.”* (Subject 12, Male).

According to the interviewees, the design of the robot is explored within a small and cohesive team, and the product is then outsourced to be manufactured at scale when there is a final prototype: *“We worked with an external manufacturer; you need to come to them with a product, and they do design for manufacturing.”* (Subject 1, Male). The important aspect is to provide a prototype that is ‘manufacturable,’ a complex topic that depends on *“all the processes that need to happen to make something at scale.”* (Subject 1, Male).

It is important that external manufacturing companies have previous experience with building social robots or some type of technology: *“They should have done a robot before.”* (Subject 9, Male) because of reliability issues:

*“It’s a very different ‘animal’ if you’ve built hardware but without moving parts or when you actually need to move. Here, you get into the reliability issues and how you build something that does not hurt the user but at the same time is robust enough. You need to find someone that has experience with hardware, robots, or mechatronics products somehow.”* (Subject 1, Male)

Besides using external manufacturing companies to build the robot, other team roles were outsourced, such as artists, *“We had several contracted animators who also helped with designing and animating some eyes.”* (Subject 4, Male); public relations team members, *“This is related to how you interact with customers; that’s pretty important, and you will want to keep this in mind as well.”* (Subject 1, Male); and marketing, *“Marketing dealt with public relations, mostly. We had a third-party public relations firm that worked with us a lot.”* (Subject 7, Female). There were some discussions about what types of team roles should not be outsourced; for example, according to Subject 1 (Male), *“I recommend not outsourcing anything that is software; that’s something you have to be building, and it is important to be in-house.”*

#### 4.3.3 International Personnel for Manufacturing

With few exceptions, the majority of the teams hired external manufacturing companies to build social robots: *“Our internal hardware team did some electrical and mechanical engineering, but we also interfaced with the various contractors. Flew to China a whole lot.”* (Subject 7, Female). According to Subject 10 (Male), *“We actually had a very good relationship with a manufacturing plant in China, and they brought another piece of, like, what can you actually build and scale.”* The idea that building a social robot requires joining the forces from multiple disciplines is highlighted in this quote:

*“We’re working with The Netherlands, Germany, the States, Australia, Japan, China. I mean, it’s an international project.”* (Subject 3, Female)

Some companies had no problem working with international teams, *“Japan, Taiwan, we worked with them to build it. The distance was not a problem.”* (Subject 9, Male). Other teams struggled to find the balance between team cohesion and long-distance professional relationships:

*“While it is great to be working across distances, it was rather difficult to coordinate the development of this robot, especially due to differing time zones.”* (Subject 4, Male)

This was supported by others:

*“I was never in China myself, but my impression of that is that to get things the way we wanted them, there had to be that very tight on the ground interaction [between the external manufacturing company and the core team]. To have someone there, keeping eyes on things and stop(ping) it from going in the wrong direction.”* (Subject 10, Male)

The main challenge with international teams was to develop solid and sustainable relationships: *“It was hard to organize times for direct communication through video conferences, and getting timely responses before deadlines was difficult.”* (Subject 4, Male). Inter-team synchronicity and mutual understanding was important because *“Manufacturing companies have their own team, and we want someone representing our company sitting in the meeting.”* (Subject 10, Male) so that both views are represented.

Different strategies were used by the teams to build a coordinated relationship across the globe. One way teams tried to synchronize was to go on-site to the manufacturing company, *“They [the engineers] would frequently go to the factory for …weeks at a time when some [robot] units were coming off. They would … just go to China for …3 weeks at a time and …stay there and intensively watch stuff roll off the line and tweak the process.”* (Subject 6, Male). However, there can be instances where a team member going on-site is impossible, so another solution was raised: *“We’d probably want to have at least an independent agent in China. That could be either someone in your own staff or you need to hire someone in China who reports to you and not the manufacturing company.”* We note that most of the external manufacturing companies referred during this study were located in China chiefly because they have the *“skills and knowledge for making things super for us, like, really lowering the cost.”* (Subject 12, Male).

## 5 Design Implications

Throughout this paper, we exposed different approaches to creating robots. Given the knowledge gathered, we now synthesize our findings into design recommendations for new robots. These recommendations map different design processes to different ways users are brought in the design of social robot. Additionally, we elaborate on the opportunities and challenges of the approaches (see Table 2). We hope to inspire future robot creators to alter their design pipelines accordingly.

**TABLE 2:** Synthesis of recommendations when designing new social robots.

Design process	Role of the user	Opportunities	Obstacles
Iterative	User is part of the workflow	Flexible design process consists of improvement loops in the robot considering users’ feedback	Design process can be chaotic, time consuming, and requires access to multiple users
Linear	There is minimal user involvement	Design process is well-defined and concrete. This can lead to faster results since it is easy to define cost, stages, and time	Design process is rigid, which can lead to undesirable results as it lacks iteration
Data points	User is a theoretical construct	This is an economical design process since it leverages accumulated expert knowledge or generalization	Risks include stereotyping the user and excluding non-traditional populations

### 5.1 Robot Design With Human in Mind

Throughout this paper, we surfaced the benefits of users’ involvement in the design of social robots. We consider it equally important to explicitly voice the negative impacts that *non-human-centric* robot design processes can have. A *non-human-centric design process* refers to minimal or non-existent user/customer involvement in the process of robot design. Designing robots without users in mind can lead to stereotype propagation, creation of erroneous assumptions about what the users need are, over-generalization and misinterpretation of problems, and other forms of bias, such as the creation of solutions that do not fit the user’s ecosystem (Benjamin, 2019). If robots are not designed with humans in mind, they can rarely succeed in helping to solve a real need or problem, falling short in the market since consumers avoid investing in expensive products (such as robots) that do not help them in concrete ways. In this work, we argue that one of the most powerful ways to counteract biases in social robot design is to follow design justice practices by creating a design pipeline that is human-centered (Costanza-Chock, 2020). By doing so, robot creators can translate human values, voices, and needs into actionable design decisions for the robotic products they are creating.

### 5.2 Distinctiveness of Social Robot Design

Designing social robots is a unique process that may not apply to other technologies. Many theories and practices from the field of human-computer interaction (HCI) need to be considered in this process. For example, designing robots underlies an iterative process, based on human needs, that requires technical precision. While designing social robots shares aspects with HCI, there are unique features of this design process that are specific to HRI. A key insight from this work is that building a robot is, in some ways, equivalent to the complexity of building an artificial being. When designing social robots, we must account for variables such as robot personality, artificial emotion expression, conversational abilities, and movement/gestures. For example, a robot can have varying levels of expressivity; they are actuated and communicate through movement to change the physical world we live in; and they are almost always anthropomorphized to a certain degree. Thus, when determining a robot’s “purpose,” it is essential to consider the combination of these variables, which cause problems unique to social robot creation.

### 5.3 Variable Alignment for Successful Robots

While it was not the goal of this qualitative study to identify and correlate variables associated with successful/unsuccessful robot products, we highlight different processes and strategies used by robot creators to better understand the challenges of building robots. Besides defining the purpose of the robot, one of the most important variables for success, our work showed a set of other variables that must be aligned. These variables include working in interdisciplinary teams, relying on outsourced labor to scale the product across time, and establishing effective international professional relations. A deeper understanding of these variables made clear that robot success in the consumer market is related not only to units of sale, but to the alignment of a complex set of variables that come into play long before the robot first appears on market shelves.

### 5.4 Additional Aspects on Social Robot Design

This work uncovered additional information about the design process of social robots. Despite going beyond the proposed research questions, we include this information as it can influence how future robot creators conceive designing a robot.

#### 5.4.1 Success Through Purpose

It is essential to have a concrete answer to the question, “Why are we building this robot?” As a participant mentioned, *“You should start with a need and provide a solution. You can say ‘I have a robot’ and then look for the problem you want to solve, but I don’t think this strategy is effective.”* (Subject 1, Male).

Being able to quantify how successful a robot is important and directly tied to the robot’s purpose. In a university lab, success might be measured by the learning gains of students or the publication record using the created robot. In a market context, success might be evaluated per unit sales or number of customer complaints. Defining metrics of success lets us circle back to the question of “Why are we building this robot?” and evaluate whether the initial purpose for robot creation is being successfully met.

#### 5.4.2 For Accuracy, Double Everything

In a study that evaluated time predictions for a coding task, results showed that programmers take 1.5x more time than initially expected, showing how “We are the worst at completing a task in the originally planned amount of time.” (Brauer, 2021). Our study shows that this is true even for expert robot creators. As one participant mentioned, *“It sounds simple, it is like, oh yeah everything is here, we have all the things, but like getting something to actually work and be reliable for a long time and also keeping the knowledge of the complex system if they don’t document, test things very, very rigorously, which is next to impossible.”* (Subject 2, Male).

A major pain point identified during our study was the underestimation of effort and cost inherent in creating a social robot, which can lead a company to fail in meeting important deadlines and initially agreed upon business goals. As a participant mentioned, to be successful *“double is the metric. Double everything: time, costs, everything.”* (Subject 11, Male). to address this design implication, it is crucial to identify the exact robot functionalities to combat the tendency to add unplanned features. In social robot design, it is easy to lose track of the initial vision for the robot when new insights and feedback are being delivered by users testing it. We argue that instead of designing for features, robot builders could adopt the approach of *designing for meaning*. Towards this end, users’ preferences should drive robot design decisions in meaningful ways, keeping in mind the original purpose for the robot.

#### 5.4.3 Simplicity in Design, Robustness in Function

The combination of simplicity and robustness in a robot are two design values that matter for its success. As participants mentioned, *“If I had to build a robot, I’d build a waaaay less complex and smaller robot.”* (Subject 8, Female), and *“It is preferable [to build a robot] that is simple, niche, and that can be done well.”* (Subject 1, Male). Simplicity has been a core design principle adopted in many ways in the design of technologies (Chang et al., 2007); it avoids anything getting in the way of the user and is defined in design as the *lack of obstruction* (Karvonen, 2000). However, most current robots fall into the category of humanoid robots, with highly complex features that run counter to the principle of simplicity. Additionally, humanoid robots frequently fall prey to the *uncanny valley* effect, i.e., feelings of uneasiness towards a robot that looks like a human but is not really human, which could be avoided by taking a simpler design approach that allows for more robustness (Mori et al., 2012).

Striving for simplicity, not only in terms of hardware but also in terms of robot identity and behavior, seems important when creating a robot. In this sense, the robot should pass a clear message to the user about what it is, what it can do, and how it will behave. Given this work, we argue that this can be conveyed in a robot through its aesthetics, such as its materiality, colors, and motions.

## 6 Conclusion

This work shed light on the design, development, and testing processes of creating social robots. The main goal of this qualitative investigation was to provide in-depth insights about building robots as marketable products that work in the real world. We have shown the existence of several layers in the design of robots: from different development processes, to several degrees of user involvement, to the complexity of team compositions. All things considered, creating robots is an extremely complex process that requires the alignment of many variables to result in a successful and lasting market product. It is thus important to question and consider the value of a robot in our lives and its place in the socio-technological world we live in and the future we want to create.

## Data Availability

The datasets presented in this article are not readily available because this was not part of our IRB. Requests to access the datasets should be directed to PA-O, patri@uw.edu.

## Footnotes

1 Statista: https://www.statista.com/statistics/755677/social-and-entertainment-robot-sales-worldwide/.

2 Robot Report ‘Remembering robotics companies we lost in 2019’: https://www.therobotreport.com/robotics-companies-we-lost-2019/.

3 Zoom: https://zoom.us.

4 Miro: http: miro.com.

5 Consumer Electronic Show (CES) is an annual trade show organized by the Consumer Technology Association (CTA) that typically hosts presentations of new products and technologies in the consumer electronics industry. Link: http://ces.tech.
